# Endothelial function and insulin sensitivity during acute non-esterified fatty acid elevation: Effects of fat composition and gender

**DOI:** 10.1016/j.numecd.2015.03.004

**Published:** 2015-06

**Authors:** K.J. Newens, A.K. Thompson, K.G. Jackson, C.M. Williams

**Affiliations:** a Hugh Sinclair Unit of Human Nutrition, Department of Food and Nutritional Sciences, University of Reading, Reading RG6 6AP, UK; b Institute for Cardiovascular and Metabolic Research, University of Reading, Reading RG6 6AS, UK

**Keywords:** Flow-mediated dilatation, Insulin signalling, Nitric oxide, Hyperinsulinaemic-euglycaemic clamp, Fatty acids, bw, body weight, eNOS, endothelial nitric oxide synthase, ET-1, endothelin-1, FAME, fatty acid methyl ester, FFM, fat-free mass, FMD, flow-mediated dilatation, iAUC, incremental AUC, LC, long-chain, NEFA, non-esterified fatty acid, NO, nitric oxide, NOx, total nitrites, PI3K, phosphoinositide 3 kinase, SFA, saturated fatty acid, SI, insulin sensitivity, TG, triglyceride

## Abstract

**Background and aims:**

We have reported that adverse effects on flow-mediated dilation of an acute elevation of non-esterified fatty acids rich in saturated fat (SFA) are reversed following addition of long-chain (LC) *n-3* polyunsaturated fatty acids (PUFA), and hypothesised that these effects may be mediated through alterations in insulin signalling pathways. In a subgroup, we explored the effects of raised NEFA enriched with SFA, with or without LC *n-3* PUFA, on whole body insulin sensitivity (SI) and responsiveness of the endothelium to insulin infusion.

**Methods and results:**

Thirty adults (mean age 27.8 y, BMI 23.2 kg/m^2^) consumed oral fat loads on separate occasions with continuous heparin infusion to elevate NEFA between 60 and 390 min. For the final 150 min, a hyperinsulinaemic-euglycaemic clamp was performed, whilst FMD and circulating markers of endothelial function were measured at baseline, pre-clamp (240 min) and post-clamp (390 min). NEFA elevation during the SFA-rich drinks was associated with impaired FMD (P = 0.027) whilst SFA + LC *n-3* PUFA improved FMD at 240 min (P = 0.003). In males, insulin infusion attenuated the increase in FMD with SFA + LC *n-3* PUFA (P = 0.049), with SI 10% greater with SFA + LC *n-3* PUFA than SFA (P = 0.041).

**Conclusion:**

This study provides evidence that NEFA composition during acute elevation influences both FMD and SI, with some indication of a difference by gender. However our findings are not consistent with the hypothesis that the effects of fatty acids on endothelial function and SI operate through a common pathway.

This trial was registered at clinical trials.gov as NCT01351324 on 6th May 2011.

## Highlights

• The impact of raised NEFA on endothelial function and insulin sensitivity was studied.
• NEFA elevation during the SFA drink reduced FMD while SFA + LC n-3 PUFA improved FMD.
• Men had a 10% higher SI with SFA + LC *n-3* PUFA than SFA, with SI similar in women.
• Changes in FMD were not mirrored by changes in circulating NOx.
• Gender mediated the effect of NEFA composition on both endothelial function and SI.

## Introduction

Non-esterified fatty acids (NEFA) have been proposed to be a mediator of insulin signalling defects in both skeletal muscle and endothelial tissue [1], [2]. Elevation of NEFA in healthy subjects by co-infusing Intralipid (a commercial lipid preparation) and heparin has been reported to impair glucose uptake and the phosphoinositide 3 kinase (P13K) signalling pathway in skeletal muscle [3], [4], [5], as well as reduce endothelial function. This pathway in endothelial cells regulates vascular tone via activation of endothelial nitric oxide synthase (eNOS) with production of the vasodilator, nitric oxide (NO). Lind et al. reversed the NEFA-induced impairment of forearm blood flow in response to methacholine [5] by infusion of insulin, supporting the notion that elevated NEFA impair endothelial function via induction of insulin resistance in this tissue. Dietary fat quality may be a contributory factor in both impaired insulin sensitivity [6] and endothelial function [7]. *In vitro* studies report more adverse effects of saturated (SFA) than unsaturated fatty acids on the endothelial PI3K insulin signalling pathway and NO production [8], [9], [10]. In human studies, the impact of SFA is less clear, however, chronic supplementation with the long chain *n-3* polyunsaturated fatty acids (LC *n-3* PUFA) found in fish oil has been consistently shown to improve endothelial function in a variety of populations [11], [12], [13]. We have previously reported that adverse effects of acute elevation of NEFA rich in SFA on flow-mediated dilatation (FMD) are reversed following addition of LC *n-3* PUFA [14]. Here using an experimental protocol, we test the hypothesis that SFA and LC n-3 PUFA differentially affect both whole body insulin sensitivity (SI) and the responsiveness of the endothelium to insulin infusion. For this study, we chose to focus on the eNOS Glu298 subgroup only, thereby excluding subjects carrying the less common allele, and providing a more homogeneous and representative population for carrying out this intensive experimental investigation.

## Methods

### Study population

From a larger cohort genotyped for a common polymorphism in the eNOS gene (rs1799983, Glu298Asp) [15], fifteen males and fifteen females homozygous for Glu298 were matched for age (mean ± SD, 27.8 ± 11.9 y) and BMI (23.2 ± 3.0 kg/m^2^). All subjects were healthy non-smokers who were not taking greater than 1 g eicosapentaenoic acid (EPA) and docosahexaenoic acid (DHA) per day, or any medication known to influence blood clotting, lipids or blood pressure. The subjects were screened for fasting cholesterol (mean ± SD 4.62 ± 0.76 mmol/L), triglyceride (TG) (1.06 ± 0.28 mmol/L) and glucose (5.15 ± 0.64 mmol/L). Subjects were recruited between March 2009 and January 2010.

### Study design

This was a single-blind crossover study; subjects attended the Hugh Sinclair Unit of Human Nutrition on two occasions separated by four weeks for females (to control for possible effects of the menstrual cycle on FMD) or at least one week for males. Subjects were randomly assigned to one of the fat loads on each day using an online number generator. Investigators responsible for performing and analysing the FMD and insulin clamp measures were blinded to the allocation and were not involved in the preparation or serving of the fat loads.

### Protocol

The study protocol has been described elsewhere [14]. Briefly, on each study day participants arrived fasted and following a baseline FMD measurement, a cannula was inserted at the wrist for venous blood sampling. A bolus fat load (66 g) was consumed at 0 min, followed by smaller volumes (22 g) every 30 min for a further 390 min. At 60 min, a second cannula was inserted into the antecubital vein in the sampling arm for the infusion of heparin. A bolus of heparin (500 IU) was followed by a continuous infusion (0.4 IU/kg body weight/min) for the remainder of the study day. At 240 min, a 150 min hyperinsulinaemic-euglycaemic clamp was performed; both insulin and glucose were co-infused into the same cannula as the heparin. Measurements of FMD were also performed immediately prior to (240 min) and at the end (390 min) of the insulin clamp.

The procedures followed in the current study were in accordance with the ethical standards of the University of Reading Research and Ethics Committee. Written informed consent was obtained from all subjects.

### Test drinks

Oral fat loads were prepared according to bodyweight (Table 1) using palm stearin (AarhusKarlshman Ltd, UK) with or without the addition of DHA-rich fish oil (Croda Healthcare, UK), 30 g skimmed milk powder (Premier International Foods Ltd, UK), 15 g chocolate powder (The Spanish Chocolate Co Ltd, UK) and 0.5 g monoglyceride emulsifier (Danisco, Denmark). Water was added to achieve a final weight of 352 g. The SFA and SFA + LC n-3 PUFA test drinks were identical in protein (11.2 g) and carbohydrate (27.1 g) content.

**Table 1:** Formulation of the test drinks.

	SFA	SFA + LC *n-3* PUFA
Palm stearin (g/kg bw)^a^	0.75	0.65
Fish oil concentrate (g/kg bw)^a^	–	0.1
*Composition of oils (%)*		
Palmitic acid; 16:0	59	51
Stearic acid; 18:0	5	4
Oleic acid; 18:1 n-9	28	24
Linoleic acid; 18:2 n-6	6	5
Arachidonic acid; 20:4 n-6	–	0.3
Eicosapentaenoic acid; 20:5 n-3	–	1.2
Docosapentaenoic acid; 22:5 n-3	–	0.4
Docosapentaenoic acid; 22:5 n-6	–	0.7
Docosahexaenoic acid; 22:6 n-3	–	10.4

a A 70 kg individual would receive 53 g palm stearin or 46 g palm stearin +7 g fish oil concentrate.

### FMD

FMD of the brachial artery was measured by trained researchers using an ATL Ultrasound HDI5000 broadband ultrasound system (ATL Ultrasound, Bothell, Washington) and a procedure based on standard guidelines, as previously described [16]. Briefly, following baseline imaging, a blood pressure cuff was inflated to 220 mm Hg to occlude blood flow for 5 min. Analysis of the images was performed using wall-tracking software (MIA-llc). FMD response was calculated using change from baseline to peak diameter divided by baseline and reported as a percentage value.

### Hyperinsulinaemic-euglycaemic clamp

Venous blood glucose was sampled immediately prior to the commencement of the hyperinsulinaemic-euglycaemic clamp at 240 min [17] to provide the target concentration for the duration of the clamp, before insulin (Actrapid, Novo Nordisk, Copenhagen, Denmark) was infused at 100 mU/kg body weight (bw) for the duration of the 150 min clamp. At 2 min, 20% (w/v) dextrose infusion was initiated, the rate of which being determined by blood glucose which was analysed at 5 min intervals (HemoCue Glucose 201^+^, HemoCue AB, Ängelholm, Sweden). The steady state glucose infusion rate over the final 30 min of the clamp provided an index of whole body SI and was expressed as mg · min^−1^ · kg^−1^ of fat-free mass (FFM). FFM was measured using a bioimpedance device (BC 418 MA, Tanita Europe, Amsterdam, The Netherlands).

### Biochemical measures

Venous blood samples were collected every 30 min into K3 EDTA (for NEFA, ET-1, insulin and C-peptide) or serum tubes (TG, NOx). To limit *in vitro* lipolysis, the EDTA samples were placed immediately on ice and processed within 30 min [18]. For analysis of C-peptide, 500 KIU of apoprotinin (Fisher Scientific, Loughborough, UK) was added per ml plasma to protect against proteolysis. NEFA and TG were quantified using an ILAB 600 (Instrumentation laboratory, Warrington, UK) with kits by Alpha Laboratories (Eastleigh, UK) and Instrumentation Laboratory respectively. ET-1 was measured by ELISA (R&D systems Europe Ltd, Abingdon, UK) and NOx using a NO quantification kit (Actif Motif, Rixensart, Belgium).

Plasma C-peptide and insulin were quantified using a multiplex assay system (Luminex 100, Invitrogen, Paisley, UK) with a Milliplex Endocrine Panel (Millipore Corp, Watford, UK). NEFA composition analysis was performed by extracting lipids from 800 μl of plasma collected at baseline (the two baseline samples were pooled) and 240 min [14].

### Statistical analysis

At 95% power and 5% significance, the minimum number of subjects required to detect a difference of 1.5% in FMD response between the two oral fat loads was calculated to be 22. Additional subjects were recruited (n = 30) to allow for possible dropouts.

SPSS version 17.0 (SPSS Inc., Chicago) was used for all statistical analyses. Summary measures calculated for the time-course data included area under the curve (AUC) and incremental AUC (iAUC). Data were tested for normality; it was necessary to log transform NEFA and TG values and use non-parametric tests for the fatty acid composition of NEFA. Independent and paired t-tests (or non-parametric equivalent) were used to compare baseline and summary measures between genders and fat loads, respectively. For postprandial time-course data, repeated measures ANOVA were performed using a mixed model approach. Bonferroni correction was applied to control for multiple pair wise comparisons. P ≤ 0.05 was considered significant.

## Results

The fat loads were well tolerated by the subjects. Initial analysis of the results revealed some differences by gender; therefore data are also presented separately for males and females where appropriate.

### Insulin sensitivity and markers of insulin metabolism

There were no differences in fasting values or metabolic responses as measured by iAUC for insulin, C-peptide, or C-peptide: insulin ratio (a marker of insulin clearance) by fat load or gender (Table 2). Males had a 10% higher SI during the SFA + LC *n-3* PUFA compared to SFA regime (P = 0.041) whereas SI was similar in females between the two fat loads (P = 0.420).

**Table 2:** Insulin sensitivity and measures of insulin metabolism.

	Whole group		Males		Females	
	SFA	SFA + LC *n-3* PUFA	SFA	SFA + LC *n-3* PUFA	SFA	SFA + LC *n-3* PUFA
SI (mg · min^−1^ · kg^−1^ FFM)	8.33 ± 0.50	8.58 ± 0.42	8.51 ± 0.53	9.41 ± 0.56*	8.16 ± 0.86	7.74 ± 0.47
Insulin						
Fasting (pM)	89.5 ± 10.3	79.7 ± 8.6	92.7 ± 16.7	78.7 ± 13.5	86.4 ± 12.8	80.6 ± 11.5
iAUC (μM/L × 240 min)	7.61 ± 1.06	8.21 ± 1.63	6.86 ± 1.43	7.55 ± 2.70	8.35 ± 1.59	8.87 ± 1.93
C-peptide						
Fasting (pM)	503 ± 36.5	476 ± 37.8	463 ± 43.2	419 ± 37.1	542 ± 58.5	532 ± 63.4
iAUC (μM/L × 240 min)	38.0 ± 5.7	31.3 ± 6.1	34.8 ± 7.0	32.7 ± 8.2	41.2 ± 9.1	29.8 ± 9.4
C-peptide: insulin ratio						
Fasting	7.1 ± 0.9	7.7 ± 1.1	6.6 ± 1.5	7.1 ± 1.3	7.7 ± 1.2	8.4 ± 1.9
iAUC	−207 ± 119	−393 ± 181	−243 ± 222	−239 ± 195	−172 ± 100	−546 ± 309

Data are presented as mean ± SEM; SI, insulin sensitivity. A significant difference from SFA is notated by * (P < 0.05). Outcome measures were available for a minimum of twelve males and twelve females.

### Endothelial function

FMD response and circulating markers of endothelial function are shown in Table 3. There was no significant difference in velocity, flow, or shear rate between fat loads at baseline or at the end of the study period (data not shown). At baseline, males had a significantly lower FMD response than females (−29%, P = 0.03).

**Table 3:** Measures of endothelial function from baseline to 240 min.

	Whole group		Males		Females	
	SFA	SFA + LC *n-3* PUFA	SFA	SFA + LC *n-3* PUFA	SFA	SFA + LC *n-3* PUFA
FMD (%)						
Baseline	5.36 ± 0.48	5.23 ± 0.46	4.35 ± 0.50	4.44 ± 0.43	6.37 ± 0.74	6.01 ± 0.76
Δ 240 min	−0.62 ± 0.27†	0.73 ± 0.23*†	−0.86 ± 0.31†	0.51 ± 0.36*	−0.39 ± 0.43	0.94 ± 0.27*†
NOx (μM)						
Baseline	24.0 ± 1.4	23.3 ± 1.1	24.3 ± 2.3	22.2 ± 1.4	23.7 ± 1.4	24.3 ± 1.6
Δ 240 min	−3.8 ± 0.7†	−3.1 ± 0.9†	−4.6 ± 1.0†	−2.4 ± 0.9†	−3.0 ± 0.8†	−3.9 ± 1.6†
ET-1 (ng/ml)						
Baseline	1.07 ± 0.09	1.04 ± 0.09	1.15 ± 0.14	1.15 ± 0.12	0.99 ± 0.12	0.92 ± 0.12
Δ 240 min	−0.07 ± 0.08	−0.06 ± 0.07	−0.05 ± 0.10	−0.02 ± 0.08	−0.09 ± 0.12	−0.11 ± 0.11

Data are presented as mean ± SEM. FMD; flow-mediated dilatation; NOx, total nitrites; ET-1, endothelin-1. A significant difference from SFA is denoted by * whilst a significant difference in the measures of endothelial function from baseline is notated by † (both P < 0.05). Outcome measures were available for a minimum of fourteen males and fourteen females.

#### Impact of NEFA elevation (0–240 min) on FMD response and circulating markers of endothelial function

For the group as a whole, compared with baseline, the SFA load resulted in an impairment (P = 0.027) whilst SFA + LC *n-3* PUFA improved (P = 0.003) the FMD response at 240 min. The mean absolute difference in change from baseline between the two fat loads was 1.35 ± 0.22% (P < 0.001). There were some indications of differential effects by gender for the change in FMD following the fat loads (Table 3); the beneficial effect of SFA + LC *n-3* PUFA was significant in females (P = 0.004) but not males (P = 0.179); conversely the impairment of FMD associated with the SFA load was evident in males (P = 0.017) but not in females (P = 0.387). Serum NOx declined to a similar extent during both fat loads (P < 0.001) and did not differ by gender. Plasma ET-1 did not change during either fat load in males or females.

#### Impact of insulin infusion on FMD and circulating markers of endothelial function (240 min–390 min)

**Figure 1:**
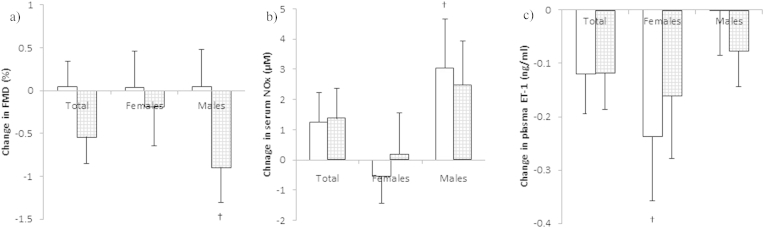
Change in a) FMD, b) Serum NOx and c) Plasma ET-1 after insulin infusion (240 min–390 min) in females (n ≥ 13) and males (n ≥ 13) following consumption of SFA (white bars) and SFA + LC *n-3* PUFA (grey bars). Data are presented as mean ± SEM. There was no difference in any measure between fat loads, significant differences within a gender group is denoted by ^†^ (P < 0.05).

In males, insulin infusion significantly reduced the post fat load FMD value by 0.89 ± 0.41% (P = 0·049) during the SFA + LC *n-3* PUFA regime (Fig. 1a). The decrease in NOx observed between 0 and 240 min was also reversed following insulin infusion in males only (Fig. 1b); this was only statistically significant during SFA (P = 0.017). For females, there was no effect of insulin infusion on FMD or NOx for either fat load (Fig. 1a and b). For females only, insulin infusion was associated with a decrease in plasma ET-1 (Fig. 1c), with statistical significance only reached during SFA (P = 0.044); no effect was seen in males.

In the group as a whole, there were no significant differences in FMD or circulating markers of endothelial function after the insulin infusion (390 min) for either fat load.

### Serum NEFA and plasma TG

**Figure 2:**
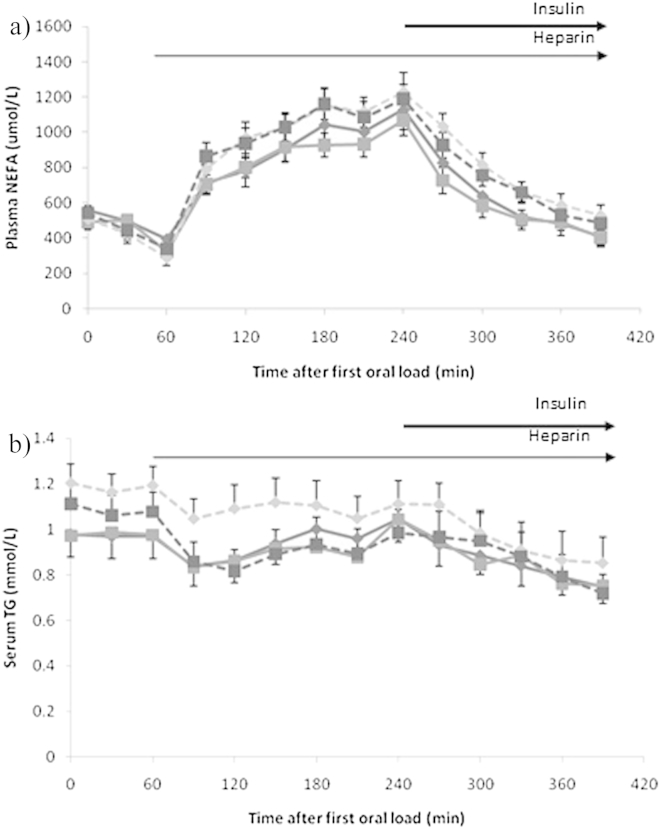
Plasma a) NEFA and b) TG following consumption of SFA () or SFA + LC *n-3* PUFA (), solid lines represent the females (n = 15) and broken lines the males (n = 14). Data are presented as mean ± SEM. For both analytes, there was a significant effect of time (P < 0.001).

Baseline NEFA and TG did not differ by fat load or gender. The oral fat-heparin protocol resulted in a two-fold elevation of serum NEFA at 240 min as compared to baseline (Fig. 2a). Concentrations of NEFA declined steadily following initiation of the insulin infusion (240 min), almost returning to baseline values at 390 min, with a significant effect of time only. NEFA response as measured by iAUC_0–390min_ was 70% greater in males than females (110.3 ± 14.2 mmol/L × 390 min vs. 64.6 ± 11.3 mmol/L × 390 min), P = 0.015.

The TG response remained within a narrow range (Fig. 2b) but was significantly higher during the SFA than SFA + LC *n-3* PUFA regime (P = 0.016). TG iAUC_0–390min_ revealed a greater reduction in TG over the study day in males (−64.4 ± 14.3 mmol/L × 390 min) than females (−20.5 ± 13.2 mmol/L × 390 min) (P = 0.029), with no difference by fat load.

### Plasma NEFA composition

There was a significant increase in the percentage weight of SFA in the NEFA fraction of plasma from baseline (median 38.8%, IQ range 36.5–40.1%) to 240 min during both fat loads (SFA; 46.0% (44.8–49.1); SFA + LC *n-3* PUFA; 43.7% (42.0–45.9); both P < 0·001). A significant increase in the proportion of LC *n-3* PUFA during the SFA + LC *n-3* PUFA load (from 1.3% (1.0–1.8) to 6.8% (5.8–7.2)) was observed at 240 min, consistent with a three-fold increase in EPA and a five and a half-fold increase in DHA (all P < 0.001).

## Discussion

We have previously shown acute ingestion of SFA with LC *n-3* PUFA to reverse impairment in FMD observed with SFA alone [14]. Findings from the eNOS Glu298 subgroup indicate that the fatty acid composition of elevated NEFA is an important factor influencing both endothelial function and insulin sensitivity. There was also some evidence of gender effects, suggesting that males were more responsive to both the negative effects of SFA on FMD response, and the positive effects of LC *n-3* PUFA on insulin sensitivity. Conversely, there were indications that females were more responsive to the beneficial effects of LC *n-3* PUFA on FMD. Differences in lipid metabolism during the protocol were also observed, with males having significantly higher NEFA concentrations than females.

Contrary to our hypothesis of a positive effect of insulin infusion on endothelial function, attenuation of the FMD response following exposure to NEFA rich in SFA did not improve after insulin infusion in the group as a whole, or in either gender. For the SFA + LC *n-3* PUFA fat load the effects observed were complex as in males, insulin infusion following this fat load was actually associated with a decrease in FMD response. As FMD has been shown to be dependent on NO bioavailability [19], it was expected these various changes in FMD responses would be mirrored by changes in circulating NOx but this was not the case in our study. Unlike the FMD response, elevated NEFA led to a reduction in circulating NOx after both fat loads, with insulin attenuating this reduction in males, but not in females. We interpret these complex findings as indicating that in the presence of elevated NEFA enriched in LC *n-3* PUFA, a reduction in circulating NOx does not result in a decrease in FMD. However, the lack of association between these two measures may be attributed to the plasma NOx measurement which represents not only NO production but also its degradation and excretion. We propose that LC *n-3* PUFA enhance endothelial function through a number of mechanisms, some of which may be independent of NO production and/or insulin signalling. For example, *in vitro* studies show that cytochrome P450 epoxygenases (CYP450) present in endothelial cells can metabolise LC *n-3* PUFA such as DHA to fatty epoxides, which promote vasodilation through activation of calcium-activated potassium channels present in smooth muscle cells [20]. Of interest to the present analysis, CYP450 enzymes have been shown to be transcriptionally upregulated by oestrogen [21]. Whether hormone dependent enhancement of CYP450 plays a role in the more marked effect of LC *n-3* PUFA on FMD observed in females in this study remains to be determined but is worthy of further investigation given the almost two fold greater response we have seen in our female subjects.

Whilst experimental elevation of NEFA has been consistently shown to impair whole-body insulin sensitivity [3], [22], [23], [24], there are very few studies which have examined the impact of NEFA composition. Decreasing the ratio of saturated: polyunsaturated fatty acids in a lipid infusion with heparin significantly improved insulin sensitivity in healthy subjects [25], whereas no difference was found in insulin sensitivity between infusions of Intralipid with and without LC *n-3* PUFA in subjects with type II diabetes [26]. In the current study, only males had a higher insulin sensitivity following SFA + LC *n-3* PUFA than SFA alone.

The sampling of venous rather than arterial or arterialised blood during the insulin clamp is a limitation of this study. With the current protocol, it was not possible to measure the primary outcome measure FMD whilst heating the hand as this has been shown to induce changes in systemic vasodilation [27]. The use of venous blood sampling is therefore a compromise but several studies do support the use of venous blood for this purpose [27], [28]. A trend for a slight decline in conduit vessel endothelial-independent vasodilatation has been previously reported during physiological hyperinsulinaemia attributed to insulin induced noradrenergic activation [29]. Due to the intensive nature of our protocol, we did not determine the dilatation of the brachial artery to glyceryl trinitrate prior to or during the insulin clamp, which could be regarded as a potential limitation of our study.

In conclusion, our study provides evidence for differential effects of SFA and LC *n-3* PUFA on FMD and on SI during acute NEFA elevation, with some indication of a difference in response by gender. We had postulated that the effects of elevated NEFA composition on impairment of FMD might reflect the differential effects of dietary fatty acids on the PI3K/Akt pathway that have been reported from *in vitro* studies [8], [9], [10]. Whilst our observations are not consistent with this hypothesis, explanation of our findings have led us to suggest novel mechanisms by which LC *n-3* PUFA may improve endothelial function and to speculate on differences in insulin dependent endothelial pathways in males and females. The clinical and public health relevance of the beneficial effects we have observed of LC *n-3* PUFA on FMD and SI are potentially significant and need to be substantiated by clarification of the underlying mechanisms involved.
